# Characteristics and Functionality of Cantilevers and Scanners in Atomic Force Microscopy

**DOI:** 10.3390/ma16196379

**Published:** 2023-09-24

**Authors:** Andrius Dzedzickis, Justė Rožėnė, Vytautas Bučinskas, Darius Viržonis, Inga Morkvėnaitė-Vilkončienė

**Affiliations:** Department of Mechatronics, Robotics, and Digital Manufacturing, Vilnius Gediminas Technical University, Plytines 25, 10105 Vilnius, Lithuania

**Keywords:** atomic force microscope (AFM), cantilever, scanner

## Abstract

In this paper, we provide a systematic review of atomic force microscopy (AFM), a fast-developing technique that embraces scanners, controllers, and cantilevers. The main objectives of this review are to analyze the available technical solutions of AFM, including the limitations and problems. The main questions the review addresses are the problems of working in contact, noncontact, and tapping AFM modes. We do not include applications of AFM but rather the design of different parts and operation modes. Since the main part of AFM is the cantilever, we focused on its operation and design. Information from scientific articles published over the last 5 years is provided. Many articles in this period disclose minor amendments in the mechanical system but suggest innovative AFM control and imaging algorithms. Some of them are based on artificial intelligence. During operation, control of cantilever dynamic characteristics can be achieved by magnetic field, electrostatic, or aerodynamic forces.

## 1. Introduction

With the rapid increase in the popularity of micro- and nanotechnologies and the growing scope of their practical application, there is an inevitable need to develop and improve the technologies and equipment required to research micro- or nanostructures. The cantilever’s design and suitability for different environments and working modes are revealed.

The first commercial model of AFM was built in 1989 and quickly became one of the most essential tools for the development of nanotechnology [1]. Typically, AFM measures a sample’s surface topology in three dimensions and studies its physical properties at the nanometric scale. Additionally, with the intensive nanotechnology development, new AFM applications were discovered [2,3,4,5,6]. The AFM control system directly impacts the quality and reliability of scientific data obtained through AFM experiments because it controls critical parameters like tip–sample interactions and feedback mechanisms, all of which influence the precision and accuracy of measurements [7].

This review focuses on widely used AFM state-of-the-art designs, reviewing cost-effective scanning speed and resolution improvement techniques and methods. From the vast amount of available information, we included in our review of papers published in the last decade that provide software or hardware improvement solutions. Priority was given to the most recent innovations with a comprehensive explanation of existing limitations, justified technical solutions, and validated modeling or experimental research results. Our review contains a detailed scientific analysis of state-of-the-art data in the field, describes the most popular AFM scanner and cantilever designs and operating modes, and comes back to the reader with a systematic approach to the cost-effective performance improvement of existing AFMs.

## 2. Background of the Atomic Force Microscope

**Figure 1:**
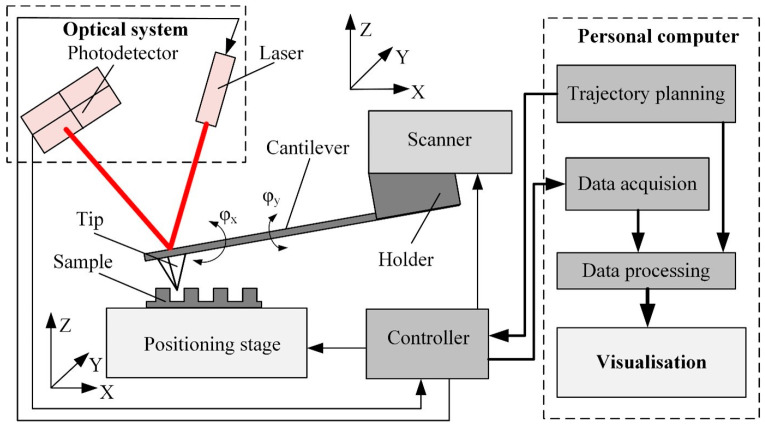
Simplified functional diagram of the AFM sensing, sample handling, and data processing system.

AFM makes use of the class of scanning probe microscopes in which the sensor contains the one-side held cantilevered probe with a nanometer-sized tip attached at the free end. AFM’s operation principle is based on keeping a constant interaction force between the tip and the sample surface when the probe moves along the sample surface (Figure 1). The tip–sample interaction force is read out as the cantilever deflection since Hooke’s law defines it as F=k·dz, where *F* is the tip–sample interaction force, *k* is the equivalent spring constant of the cantilever, and *dz* is the deflection of the cantilever. The sample’s surface topography parameters are obtained from the error signal, defined by the difference between the setpoint deflection and the actual value of *dz*. To determine the properties of sample materials, such as adhesion, friction, or viscosity, more cantilever deformation modes can be measured, for example, twist or deviation (φ_x_, φ_y_) in the horizontal plane [8].

Typically, AFM consists of a few functional blocks: a positioning stage, a cantilever, a scanner, a controller, data processing, and visualization algorithms (Figure 1). The positioning stage ensures sample movement along three orthogonal axes; it consists of a set of precision drives and guides the positioning of the sample in the x, y, and z directions.

**Figure 2:**
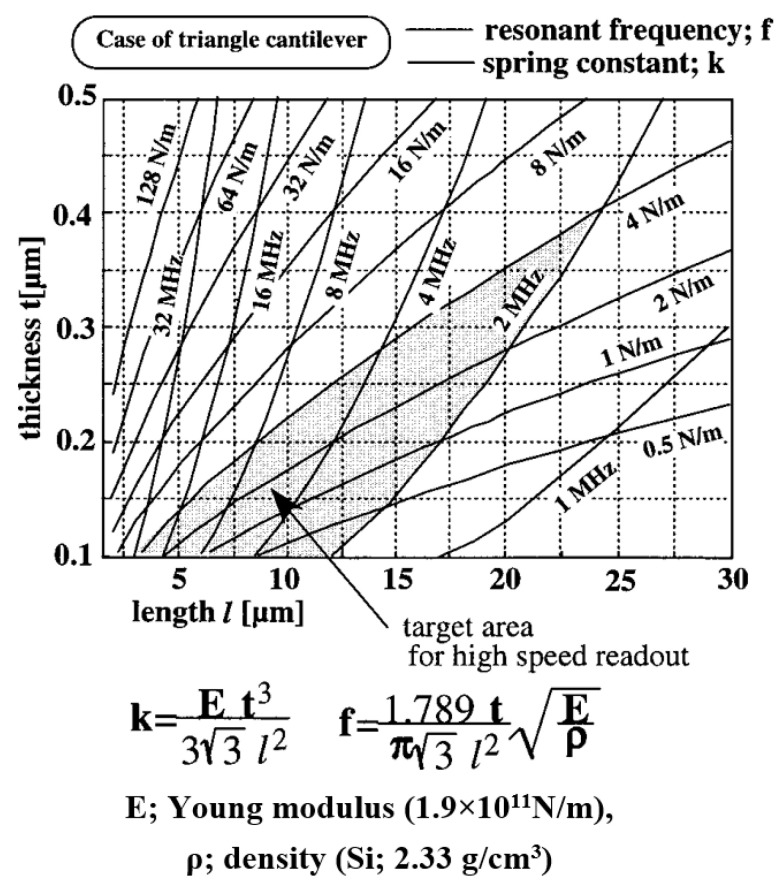
Contour map of the resonant frequency and spring constant of the AFM cantilever due to the AFM cantilever length, l, and thickness, t. Adapted with permission from [9].

The complex sensor contains a cantilever, an optical system, and a scanner. Cantilever properties (i.e., stiffness and resonant frequency) depend on the design and material properties. Dynamic cantilever characteristics directly impact measurement result reliability since these characteristics limit the maximum possible scanning velocity and resolution [9]. Cantilever tip geometry determines the AFM imaging accuracy and resolution and influences the excitation of the cantilever [10] (Figure 2).

The cantilever deflection is usually measured by an optical system consisting of a laser source and position-sensitive photodiode matrix [11]. The laser beam reflects from the upper surface of the cantilever and falls onto the active area of the photodiode matrix. The cantilever deflection is obtained from the position of the reflected laser spot. The reflection provides *z*-axis feedback to the controller. The *z*-axis deflection error signal and the tip coordinates are also transferred to the data acquisition and processing software to produce a three-dimensional image of the sample surface.

Cantilever deflection can also be measured by nonoptical methods based on capacitive, piezoelectric, or piezoresistive phenomena [12,13,14,15,16]. The deflection sampling rates generally are much higher than the settling time of the scanner [11]. Increasing the imaging speed, resolution, or both usually means increasing the sampling rate.

**Figure 3:**
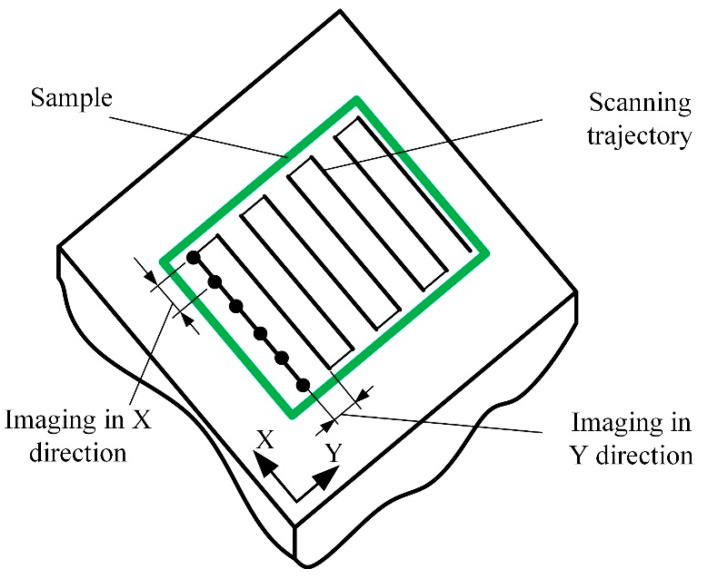
Schematic representation of the imaging process.

**Figure 4:**
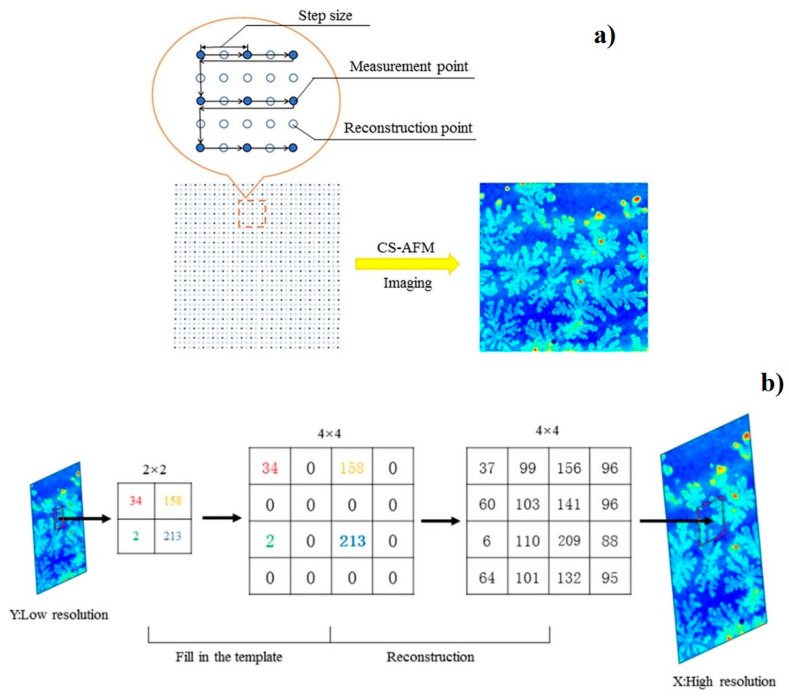
(**a**) Super-resolution AFM imaging using compressed sensing (CS). (**b**) Super-resolution imaging schematic based on compressed sensing. Adapted from [17].

Before imaging, a few scanning parameters must be chosen: the size of the field of view, the scanning mode and speed, and the number of lines and points in each line. All these parameters will affect the overall image acquisition duration and future image resolution (Figure 3). The most popular imaging strategy is to start from a sparse, large field-of-view image, possibly interpolated by AI-enabled algorithms [17]. After that, the smaller region of interest is selected for high-resolution imaging (Figure 4). The software creates the image from sampled data points using various filters and interpolation algorithms [18]. Reconstruction-based imaging techniques and “Compressive sensing” theory can be used to minimize the number of required data points and, at the same time, to ensure a high resolution of the final image [19].

Optimization of the scanning parameters is one of the paths for increased AFM performance. Usually, the optimization strategy is subject to AFM operator experience and skills, and the optimization algorithm is based on the database [20] or a mathematical model [21], taking known data, such as the expected geometrical shape and material properties of a sample and the probe geometry, and calculating optimal scanning parameters to produce the required resolution of the image within an adequate time and with a minimum of image artifacts [22].

### 2.1. AFM Operating Modes

**Figure 5:**
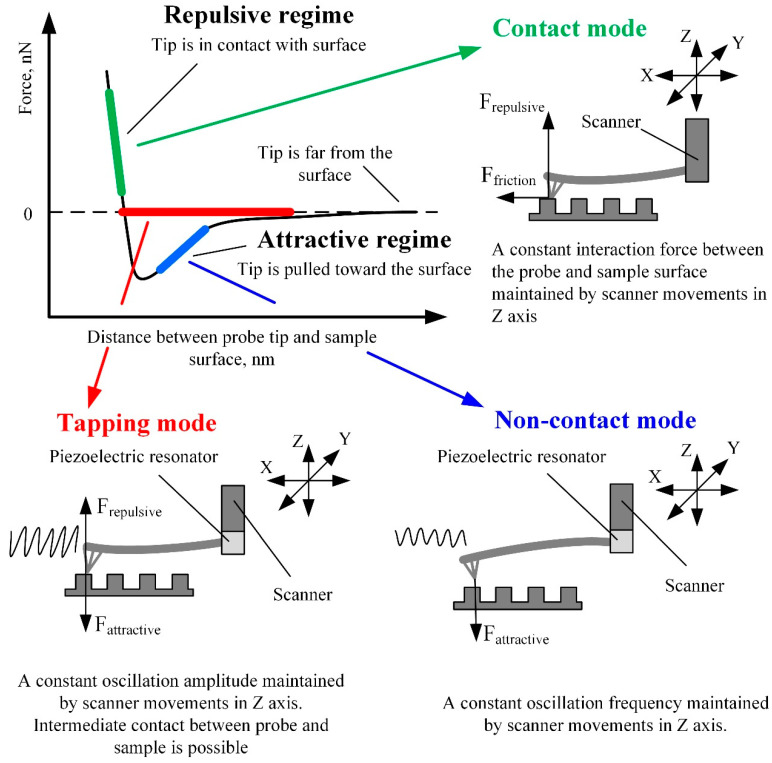
AFM working modes in respect of Van der Waals force.

Depending on the interaction between the probe and the sample surface, three main AFM operating modes can be distinguished: contact mode, noncontact mode, and dynamic contact (tapping) mode [23,24]. The Van der Waals force mainly characterizes the probe and sample surface interaction. The intensity and direction of this force strongly depend on the distance between the probe and the sample surface (Figure 5).

The contact mode of AFM is based on scanning the surface in repulsive force mode by keeping a constant 1–2 Å distance from the sample surface. In this scanning mode, cantilever stiffness is essential since it directly impacts the cantilever’s deflection amplitude. Therefore, cantilevers of lower stiffness enable the imaging of surfaces with smaller feature sizes since the higher deflection of softer cantilevers produces a more significant error signal. On the contrary, if the cantilever is stiff and the sample surface features are small, the error signal can be too small to be sensed. The advantage of the contact mode is that a thin layer of water, which is always present on the surfaces of solid samples at standard conditions, has a minor influence on the measurement results. Therefore, due to the strong surface forces, the probe is attracted to the sample surface and “pierces” the water layer [25]. The contact mode also enables the measurement of friction, conductivity, elasticity, and other material properties. Moreover, the contact mode provides a higher resolution and scanning speed than different modes and enables the examination of surfaces with a broader range of features [12,25].

The speed limit is the main disadvantage of the contact mode. The low resonant frequency of soft cantilevers is the cause. During the scanning process, the sample surface structure causes kinematically excited oscillations of the cantilever. The frequency of these oscillations depends on the scanning speed. When the cantilever’s excitation frequency approaches its resonant frequency, the contact between the probe and the sample becomes unstable, making scanning results inaccurate [26]. Another disadvantage of the contact mode is that it is unsuitable for soft materials because the probe can damage soft biological materials and polymers. While scanning inhomogeneous materials, surface forces can have uneven magnitudes, lowering the reliability of the resulting topography image [27,28,29,30].

When the cantilever tip is near the surface of interest, an attractive Van der Waals force occurs between the tip and the surface. Scanning at the distance of attractive force is called noncontact mode, and it is mainly used for the analysis of soft (biological and organic) materials. In this mode, a special piezoelectric resonator mounted on the AFM cantilever fixture place (Figure 5) kinematically excites the cantilever base, which causes the cantilever to vibrate at a frequency close to its resonant frequency. When the probe approaches the sample surface, the cantilever oscillations’ frequency decreases due to attractive forces. By analyzing the variation in cantilever oscillation frequency, it is possible to determine the surface topography, viscosity, friction, and probe–surface interacting force [23]. The noncontact scanning method is more sensitive to ambient conditions than the contact mode [23]. While operating in the noncontact mode, it is required to ensure that the layer of liquid on the sample surface is thinner than the surface forces activity range, otherwise the tip of the cantilever can seize in the liquid layer and touch the surface of the sample. Contact with the scanned surface distorts the measurement results and can damage the cantilever or the sample, especially at a high scanning speed. The abovementioned problems can be avoided in tapping (dynamic contact) mode [31].

The tapping mode lies between the contact and noncontact modes and has features of both. In this mode, the cantilever is kinematically excited by the piezoelectric resonator, similarly to in noncontact mode (Figure 5). The only difference is the excitation amplitude—it is about ten times higher than in noncontact mode and can reach up to 200 nm. When the probe approaches the surface of interest, the cantilever’s oscillation amplitude decreases due to the surface forces acting on the tip. Still, the tip can accidentally touch the sample surface. The feedback of the *z*-axis position of the AFM scanner is used to maintain the constant oscillation amplitude of the cantilever. The image is obtained by mapping the *z*-axis feedback signal, which corresponds to the variation of the tip–sample interaction force [32]. The main advantage of the tapping mode is a higher image resolution and accuracy than the noncontact mode. The tapping mode is more suitable for scanning softer materials than the contact mode and is applicable for researching delicate biological samples. The tapping mode’s main disadvantage is a lower scanning speed and accuracy than the contact mode.

**Figure 6:**
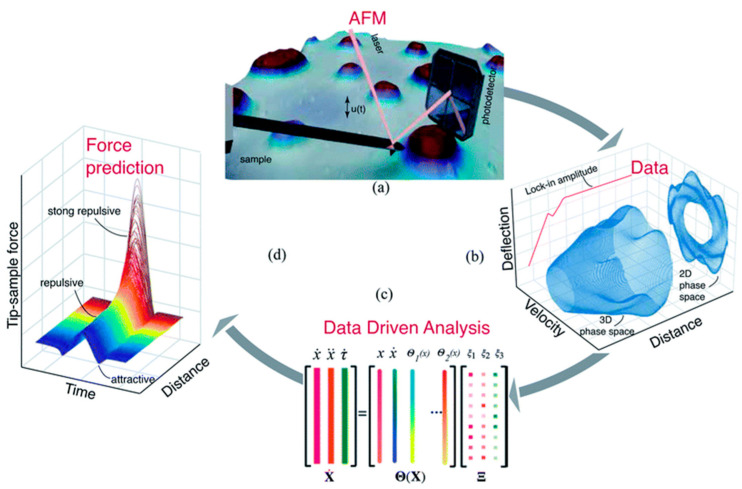
Schematic of the identification process. (**a**) Experimental data are obtained directly from the photodetector of the AFM. (**b**) The data are captured using a field programmable gated array device and post-processed to create state vector channels. (**c**) The state vectors are used as inputs in the sparse identification algorithm to discover the governing model of the system. (**d**) The data-driven model is used to estimate the tip–sample interaction force. Reproduced from Ref. [36] with permission from the Royal Society of Chemistry.

It is essential to mention other scanning modes when AFM operates in the intermediate regime between repulsive and attractive forces: peak force tapping and phase imaging. The first enables precise control of probe–sample interaction at very low interaction forces [33]. Typical peak force tapping applications include high-resolution imaging of soft samples in a liquid environment and nanomechanical and electrical/electrochemical property measurements. The phase imaging mode monitors the phase difference between the excitation and response signals of the cantilever, while oscillation amplitude is maintained constant by the *z*-axis position feedback [34]. This scanning mode is used for samples with significant surface irregularities and enables simultaneous mapping of topography and other material properties, such as adhesion, elasticity, and viscoelasticity. Phase imaging mode created the background for many other AFM techniques, such as magnetic force microscopy (MFM), electric force microscopy (EFM), and scanning capacitance microscopy (SCM) [35]. Furthermore, recent advances in machine learning allow the characterization of probe–sample interactions from experimental data with sub-microsecond resolution and open new capabilities for visualizing dynamic biological processes [36]. The first algorithm was trained on standard AFM models and then showcased experimentally with a polymer blend of polystyrene (PS) and low-density polyethylene (LDPE) sample (Figure 6).

**Figure 7:**
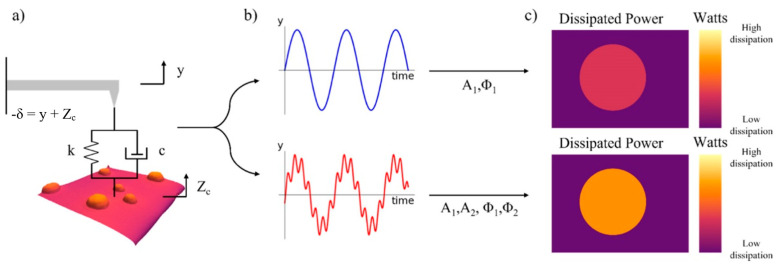
System schematic. (**a**) Cantilever–sample interaction: y is the cantilever position, *Z_c_* is the average distance between the tip and the sample, *δ* is the tip–sample gap/indentation, *k* is the tip–sample stiffness, and *c* is the tip–sample damping. (**b**) Cantilever position versus time for single/tapping mode and bimodal mode AFM. (**c**) Dissipated power images of single/tapping mode and bimodal mode AFM. Adapted from [38].

One of the prospective modes of AFM, which allows increasing imaging resolution, is multifrequency AFM, in which the cantilever is simultaneously excited/observed at two or more separate frequencies [37,38,39]. In bimodal AFM, the first two flexural cantilever modes are used (Figure 7).

### 2.2. AFM Imaging Speed Optimization and Performance Increase Methods

**Figure 8:**
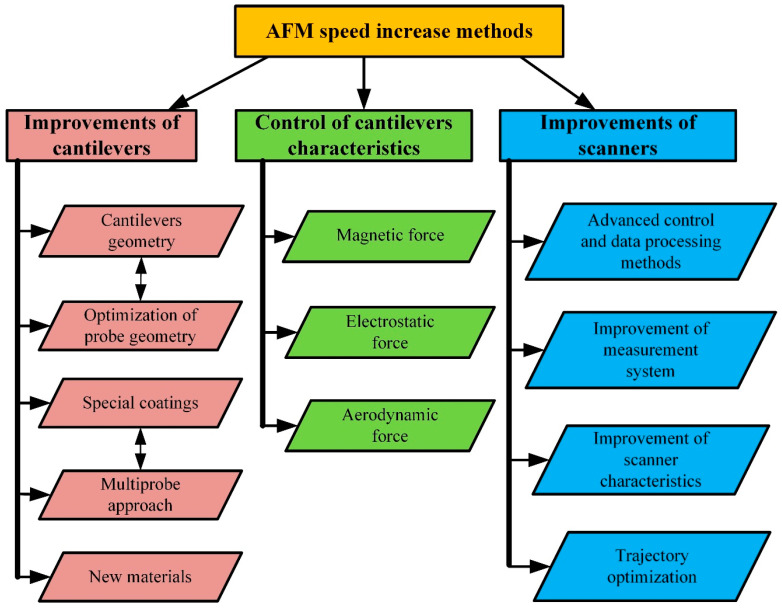
Classification of AFM scanning speed increase methods.

While scanner properties remain constant, imaging speed can be optimized by choosing the proper scan range in the *x*-axis direction, the number of scan lines, the spatial frequency of the sample surface, and the maximum possible phase delay in tracing the sample surface [40]. AFM performance can be improved by various approaches, as shown in Figure 8. There are three main performance improvement trends: improvements focused on enhancing cantilever characteristics and designs, implementations of different cantilever property modification methods, and modifications of scanners.

All the categories mentioned above contain several subcategories. The development of cantilevers focuses on the improvement of the following parameters: I—an increase in the resonant frequency of the cantilevers; II—optimization of the probe geometry; III—the application of various surface coatings for better reflectance or the production of specialized cantilevers whose deflection can be measured using the nonoptical method; IV—cantilever arrays suitable for multiprobe scanning; V—new materials for cantilever manufacturing.

The research focused on the in situ control, modification, and adjustment of cantilever characteristics is based on the hypothesis that instead of selecting and changing cantilevers for each case, it is possible to modify the dynamic characteristics of the cantilevers in situ, thus extending their field of application. This approach requires the application of external controllable force on a probe surface. This can be performed using magnetic, electrostatic, or aerodynamic force on the cantilever surface [26].

The AFM scanner improvements cover two general areas: software and hardware. Software improvement focuses on scanning trajectory optimization, the development of advanced control, and data processing methods. Hardware modernization concentrates on the enhancement of scanners and measurement systems.

In the next sub-sections, we will focus on the cantilever properties, in situ cantilever characteristic adjustments, and AFM software improvements as mostly cost-effective and highly available methods, skipping the review of radical hardware engineering approaches, such as multiprobe designs [41,42] and cantilever-free AFMs [43], since reviewing these trends would overextend the scope of the present article.

## 3. Cantilevers

The resonant frequency and bandwidth are the parameters that determine the dynamic properties of the cantilever. A wider bandwidth means more damped behavior, and a narrow bandwidth means more oscillating behavior. The idea of the AFM sensor as a dynamically sensitive mechanical system remained unchanged for several decades until the concept of cantilever-less AFM sensors came about [43]. Still, the dynamic characteristics of the cantilever can be modified by changing its geometry and dimensions as well as the cantilever and tip materials.

Modern cantilevers are typically fabricated from monocrystalline silicon (Si) or silicon nitride (Si_3_N_4_) using photolithography, etching, and other microfabrication technologies [44,45,46]. The length of cantilevers usually varies from 40 μm to 500 μm, the width reaches up to 50 μm, and the thickness varies within the range of 0.5 μm to 8 μm. The tip height usually does not exceed 10 μm, and its apex radius is several nanometers. The force constant, which characterizes the cantilever’s stiffness, ranges from 0.01 N/m to 50 N/m [47,48,49]. Probe geometry—forward, backward, side angles, and tip height—are also parameters that affect scanning resolution and limit the maximum detectable roughness of the sample surface. Moreover, tips could be coated with various materials to extend their application field [50].

Despite the considerable variety of cantilevers, the right choice remains undefined and requires an individual solution, often supported solely by the operator’s experience. Usually, cantilevers are selected according to the necessary scanning resolution, the sample’s physical and geometrical properties, a reasonable scanning speed, and the suitable scanning mode. Nevertheless, the researcher’s experience remains key to successful AFM application.

The classical vibration theory [51] suggests three tactics for the minimization of the errors caused by the dynamic response of the AFM cantilever:

- Select the probe with the appropriate stiffness, resonance frequency, and resonance quality.
- Adjust the cantilever working conditions to increase damping and shift the resonance frequency away from the selected regime.
- Select scanning parameters (scanning speed and resolution) to operate above the resonance frequency.

### 3.1. Geometry of Cantilevers and Tips

Numerous papers describe optimizing the cantilever’s resonant frequency and stiffness by changing its geometry [52,53]. The general tendency is to increase the resonant frequency without an increase in the cantilever’s stiffness. The impact of cantilever length, tip shape, and lateral contact stiffness on the sensitivity and resonant frequency of the cantilever is described in [54]. The authors report that increased lateral contact stiffness leads to higher AFM sensitivity, especially in high-order vibration modes. Also, they found out that cantilever length impacts the resonant frequency and sensitivity more significantly if the value of the standard contact stiffness is high, for example, when hard materials are measured. Higher lateral stiffness has a similar effect, and its impact becomes noticeable when standard contact stiffness increases.

**Figure 9:**
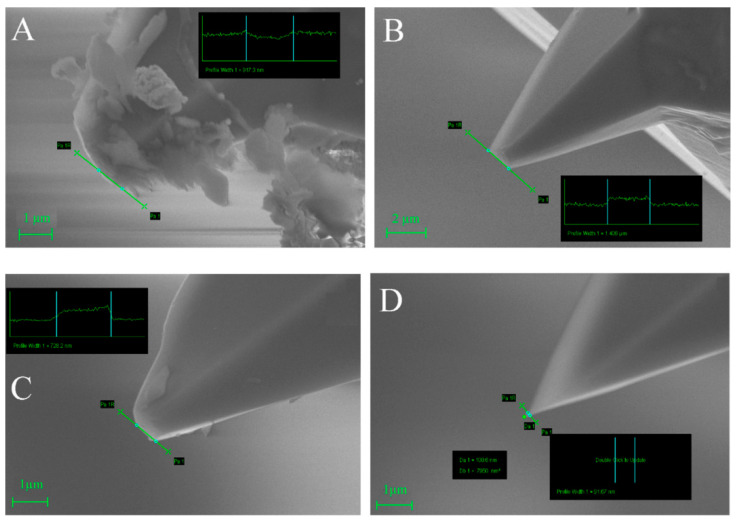
SEM images of AFM tips with radii of (**A**) 1745 nm, (**B**) 1406 nm, (**C**) 728 nm, and (**D**) 53 nm. Adapted from [22].

**Figure 10:**
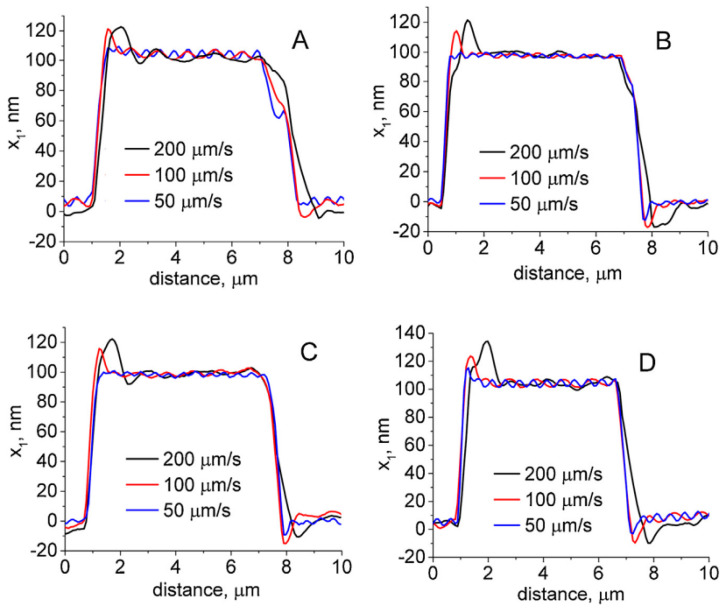
Scan of a silicon calibration grid at different scanning speeds using (**A**) a 1745 nm radius tip, (**B**) a 1406 nm radius tip, (**C**) a 728 nm radius tip, and (**D**) a 53 nm radius tip. Adapted from [22].

Experiments with differently worn tips were performed in [22] (Figure 9). The authors also developed a mathematical model to determine the quality of the picture before scanning when some parameters, such as scanning speed, the material of the sample, and tip quality, are known. Experiments with the calibration grid revealed that a contaminated tip creates adhesion artifacts (Figure 9A and Figure 10A at distances from 7 to 8 µm). A similar tip geometry without contamination shows less adhesion (Figure 9B and Figure 10B). At distances from 1 to 2 µm, the artifacts that depend on scanning speed could be seen (Figure 10A–D).

Typical cantilevers are 85–500 µm long and have resonant frequencies less than 500 kHz [55]. The paper [9] describes a method for increasing the speed of an AFM using specially made short triangular cantilevers 7–20 µm long and 0.3 µm thick with a resonant frequency of 6.6 MHz. The authors describe the manufacturing process of such cantilevers, propose a new system for cantilever deflection measurement, and provide an experimental demonstration of increased imaging speed. Shorter cantilevers are also less affected by viscous damping. Therefore, there is some potential for higher sensitivity [56,57,58]. Moreover, short cantilevers with higher resonant frequencies increase the sensitivity of the high-speed AFMs which are used for the live imaging of living cells or other biological samples that can move. Scanning speed in such cases is expressed in frames per second [59].

Ultrathin (60 nm) silicon cantilevers with force constants in the range of µN/m and resonant frequencies in the vacuum of 1.7 kHz were made for work in the air [60]. These cantilevers provide high force sensitivity and allow the maintenance of sub-0.1 nm tip–sample distances. Also, short cantilevers are used in liquid media [58,61]. The authors used ~30 µm long cantilevers for imaging biological samples in liquid. They confirmed that shorter cantilevers allow faster imaging, even if they are pretty stiff (1.3 N/m), compared to longer ones. According to [55], the best cantilevers for a liquid medium have resonant frequencies of 100–200 kHz in liquid and spring constants of 0.1–0.2 N/m.

Further improvements and optimizations of AFM cantilevers and their tip geometries will contribute to the availability of adequate photolithography and thin-film deposition/etching technologies.

We provided a method suitable for making 2–10 µm length cantilevers [56]. This method comprises fabricating a sacrificial cantilever of SiO_2_ and depositing a layer of material which will form the final cantilever on the sacrificial one. The sacrificial cantilever will be etched away. A compound cantilever fabricated from two fused silicon oxide wafers was made with a separate bending and vibrating part [62]. The vibrating part had a lower mechanical resonant frequency than the bending one.

Research on the relationship between geometric parameters and the stiffness of the triangular cantilever in the literal, standard, and longitudinal directions was performed [63,64]. The researchers defined these dependencies as mathematical equations and proposed a mathematical model that allows the determination of the cantilever’s optimal thickness using experimental resonant frequency measurement results. The results suggest that short, rigid, triangular cantilevers are best suited for high-speed imaging.

Several scientists have conducted similar studies dedicated to creating cantilevers with specific resonant frequencies by changing the geometry of cantilevers. The paper [65] presents attempts to design an AFM cantilever with a higher resonant frequency at the same stiffness. In [66], an effort was made to fabricate cantilevers with higher resonance frequencies using precise laser micromachining techniques or ion beam milling. The idea is to modify triangular cantilevers by thinning their edges with more advanced microfabrication technologies than photolithography. A finite-element model (FEM) that allows the optimization of the geometry of the cantilever and the shift of resonance frequencies relative to each other is provided in [67]. Using the proposed FEM model, the authors designed and fabricated a cantilever with the second resonant frequency in a lower frequency range. The mathematical model with a similar purpose provided in [68] allows the design of cantilevers with exactly specified ratios between the magnitudes of the first, second, and third resonance frequencies by adjusting the geometrical parameters.

The impact of tip conicality and tip apex radius on AFM accuracy was investigated in a couple of works [69,70]. The authors proposed a method to measure the tip apex radius and tip conicality by performing a test scan of a known structure. The paper [71] discloses studies of cantilevers for noncontact AFM mode. The researchers developed a cantilever with a tip length approximately equal to the cantilever’s length. The proposed mathematical model of the cantilever was used to investigate the cantilever’s dynamic characteristics concerning the long tip parameters. Relationships between cantilever dynamics and tip geometry were found [54,71].

In [72], a detailed methodology for tip characterization is provided based on sample scanning with a calibrated surface structure. Characterizing the tip enables minimization of the tip shape “footprints” in sub-nanoresolution scans. Moreover, the AFM imaging accuracy described the nonlinear tip–surface interactions occurring in tapping mode [73]. This nonlinear phenomenon is especially prominent on high-adhesion surfaces, such as polymers and biomolecules. High adhesion forces between the tip and surface act as additional damping forces, complicating the control of cantilever oscillation amplitude.

The research efforts directed toward fabricating cantilevers with a smaller tip radius are constantly growing. A smaller tip radius is necessary to increase AFM scanning resolution and accuracy. It is also essential to note that imaging error, caused by the tip radius size, increases during the process due to mechanical wearing, especially during scanning of hard surfaces in the contact mode [22,74]. The authors defined that several effects, such as abrasive wear, low cycle fatigue, and plastic deformation, could appear by scanning a silicon surface with a silicon nitride tip. Current microfabrication technologies approached tip dimension limits. Producing probes with a smaller than 1–2 nm tip radius is challenging using silicon. Therefore, tip improvements are developed by modification of the tip by gallium nitride (GaN), copper oxide (CuO) nanowires, or carbon nanotubes [75,76,77]. These nanostructures usually are about 5 μm in length and can vary between 0.4 and 40 nm in diameter. Such tips are better suited for imaging samples with more significant surface irregularities since a needle-shaped tip reduces errors caused by the tip geometry and increases the achievable scanning resolution. Also, the use of gallium nitride nanowires and carbon nanotubes has led to the development of AFM cantilevers with increased wear resistance: even after scanning several samples in contact mode, the tips of such sensors remain sharper than 150 nm [75,76]. Reduced tip mechanical wear was observed because carbon nanotubes and silicon formed a composite structure on the tip, enhancing its hardness compared to pure silicon.

Using copper oxide nanowires leads to the development of operable sensors, but their accuracy is slightly lower than that of the standard AFM sensors. The decrease in accuracy appeared to be due to the nanowire deformation during the scanning process [77]. It is also essential to note that the fabrication of the cantilever tip with nanostructures hardly keeps the repeatable geometrical parameters since there is no technology to ensure this [75].

Research on the physical parameters of cantilevers, including the shaping of the cantilever tip, targeted for improved AFM performance, indicates some pathways for possible advances beyond the state of the art, but only for narrowly specified applications [78]. The main disadvantage of cantilever/tip optimization is the impossibility of advancing significantly with cantilever/tip design to cover the needs of a wide range of practical situations. Each research case requires a unique cantilever with specific parameters optimized for this case. It is also essential to admit that cantilevers’ design, fabrication, or modification is complicated, expensive, and generally impossible without specialized equipment and related competencies. A summarized review of cantilever designs and their parameters is provided in Table 1.

### 3.2. Special Coatings and New Materials

AFM cantilevers made of silicon or silicon nitride have approached the limits of their speed/resolution capability. The production of more miniature cantilevers with sharper tips or higher resonant frequencies is challenging due to the limitations of traditional technologies [81]. Alternative cantilever materials are polymers, for example, SU-8. Polymer cantilevers have considerably higher damping coefficients than those made of silicon or silicon nitride. Therefore, a higher scanning speed in noncontact mode can be achieved; in ambient air, a 19-times speed improvement was demonstrated over similarly sized silicon nitride cantilevers.

Silicon and silicon nitride have relatively low reflectivity. Therefore, cantilevers are coated with a gold or aluminum layer for proper optical detection to increase reflectivity. This way, an unwanted temperature-sensitive bimaterial strip is created, which will unpredictably deform due to the temperature change and cause the drift of the readings. This effect becomes especially significant for miniature cantilevers since the metal layer’s thickness is comparable to the cantilever’s. If cantilevers were made only from metal, it would be challenging to control residual stress in thin metal films as it would cause most metal cantilevers to bend [57]. Adding a metallic reflector pad to the cantilever end makes it possible to maximize the reflectivity while minimizing sensitivity to temperature changes. Meta-surfaces can be integrated into cantilevers to increase optical sensitivity and reduce the number of faulty reflections that appear due to cantilever oscillations [79].

Piezoelectric coatings can be used to adjust the cantilever’s stiffness or damping parameters, increasing the scan rate and imaging accuracy in tapping mode AFM [82]. It was also shown that piezoelectric control can minimize errors caused by significant irregularities on the sample surface, such as steps and holes with straight edges. AFM cantilevers with two piezoelectric coatings create an excellent possibility for the sophisticated response to excitation. A cantilever with two piezoelectric elements in nonlinear interaction between the tip and the sample can be brought into the complex oscillation modes with improved measurement possibilities [83]. Korayem and Nahavandi [84] modeled cantilevers with two piezoelectric film layers. They researched cantilever behavior in a liquid medium for various fluid levels and sample surface features. Satoh [85] has described the possibilities of using this cantilever type in near-field scanning optical microscopy.

Piezoelectric cantilever coatings can also eliminate optical readout from the AFM, thus simplifying it. One of the most popular piezoelectric materials is zinc oxide (ZnO), which can be deposited in a thin film. Moreover, a piezoelectric coating allows the simultaneous use of a cantilever as a high-resolution nanoactuator. It has at least a two times higher sensitivity potential than the optical readout systems. Researchers showed that this method enables multiprobe imaging with several cantilevers working in parallel [86]. Another multiprobe approach contains a CCD camera and a supercontinuum laser-based optical readout system [53]. Such technology is suitable for the same cases as traditional AFM. Multiprobe solutions significantly increase the field of view and imaging speed and, combined with an intelligent trajectory-planning algorithm, were demonstrated as a solution for high-speed, large field-of-view applications. Still, systems with arrays of cantilevers are highly complex and expensive and therefore rarely used in practice.

Recent cantilever setups with piezoelectric actuators are sensitive to ambience disturbances and have high electrical isolation requirements since a comparatively high voltage is used. An isolated AFM cantilever with a piezoelectric film was designed for operating in a noncontact mode in a liquid environment [87]. This kind of cantilever is suitable for studying biological materials covered by a layer of liquid [88].

The main disadvantages of the AFM cantilevers with the piezoelectric films are as follows: deposition of piezoelectric thin films increases the overall thickness of the cantilever and introduces intrinsic stress [89]. There have been developed piezoresistive cantilevers with resonant frequencies of 6 MHz and spring constants of 2 N/m [90]. However, such cantilevers have low sensitivity and are challenging to work with in liquid media due to the increased electrical losses. Instead, they are used for nanometric-scale surface imaging in force-based magnetic resonance imaging [91].

### 3.3. Adjustment and In Situ Control of Cantilever Dynamic Characteristics

In 1994, Florin [92] proposed a high-speed AFM method based on the active control of cantilever dynamic characteristics. The interaction force between the probe and the sample surface was modified using an external magnetic field that acted on the cantilever ferromagnetic surface [92]. The magnetic force increases the scanning speed when operating in a noncontact mode due to the increased damping or shift of the cantilever resonance frequency. The main limitation of this method is the undesired interaction between the sample and the magnetic field.

Another approach is to complement the AFM technique by measuring the electrostatic force during the short-range tip–sample interaction [93]. This allows one to achieve fast micro- and nanosecond-scale imaging of cell dynamics in a noncontact mode. Research on the effect of electrostatic forces in large-range imaging (interaction forces in the range of a few piconewtons) has been carried out in [94]. The authors proved that it is possible to avoid mechanical wear of the tip and achieve high resolution by using such a regime.

During AFM operation, the magnetic field can be used for cantilever actuation. The magnetic field enables simultaneous micromachining and surface characterization processes [95]. The authors provided innovative switchable dual cantilever systems with a narrow gap between them and a magnetostrictive coating on the cantilever bottom surface.

Recently, in situ cantilever dynamic adjustment was proposed by applying aerodynamic force on the cantilever’s upper surface [26,96,97]. This was proven to be effective for a contact mode. It was demonstrated that using a laminar flow of compressed air to the cantilever’s upper surface increased the effective stiffness and reduced tip bouncing and created corresponding opportunities for increased scanning speed. Overall, the topography measurement error was reduced by 20% at a scanning rate of 847 µm/s. This modification is applicable with various standard cantilevers without creating a magnetic or electrostatic impact on the sample.

AFM cantilever actuation in fluid media can be performed by micro-electromechanical system (MEMS) acoustic transducers operated at very high frequencies of 100-300 MHz [98]. Not only the possibility of in situ cantilever dynamic adjustment by acoustic radiation pressure was demonstrated, but also in situ characterization of the cantilever spring constant and dynamics.

Even though the in situ cantilever dynamic adjustment method seems promising and gives outstanding results, it is seldom used in practical applications since it requires modifications which complex and expensive commercial AFMs can hardly tolerate. The custom modification of their design or components usually voids the warranty and makes proper commercial equipment maintenance difficult. Overall, these methods lead to increased scanning speed and improved resolution, which generally would be limited by the properties of cantilevers.

## 4. Scanners

The dynamic characteristics of AFM cantilevers are the primary factors limiting the imaging speed of commercial AFMs. Nevertheless, imaging speed is also heavily determined by the scanner design, control, and data processing algorithms. There are several trends for scanner design improvements (Figure 6), from the general increase in the scanner mechanical stiffness to the increased effective field of view. Scanner stiffness is usually ensured by reducing the number of moving, sequentially connected mechanical links. One of the ways to achieve this is to design independent *z*-axis and x–y axe drives [99]. Increased general stiffness of the scanner leads to increased imaging speed, accuracy, and field of view.

High-speed AFM with a parallel kinematic piezoelectric actuator demonstrated significantly higher resonance frequencies: 5.6 kHz for the x–y axes and 29 kHz for the *z*-axis [100]. A field-of-view increase beyond the usual 120 µm × 120 µm can be achieved by stacking several nanomanipulators and resulting in a more significant stroke [101]. The control algorithm of the extended field-of-view scanner must account for the complex dynamics of sequentially connected multiple mechanical links and adequately compensate for positioning errors. Similarly, a combination of slow, low accuracy and fast, high accuracy drives can result in improved control characteristics of the extended field-of-view scanner [92]. A commercially available positioning stage for the slow motion of the *y*-axis and fast piezoelectric *x*-axis drive allowed a scan of a 170 × 170 µm area in one second with an average tip velocity of 13–28 cm/s (or peak tip–sample rates of 20–44 cm/s). An AFM scanner with rotational scanning eliminates the delays of reciprocating movement and achieves a higher scanning speed with a larger field of view [102]. A linear scanning speed of up to 45 mm/s was demonstrated.

Sometimes, AFM performance can be improved by carefully optimizing all scanner components [103]. The research described in [11] demonstrates that an optical pickup unit from a PC DVD drive can be successfully implemented into AFM as a deflection sensor. Using the flexural stage and interferometric position measurement system, the natural frequency of the scanner x- and y-axes can be increased to 4.37 kHz and 3.75 kHz, respectively. As a result, this design achieved a scanning speed of 8 mm/s [104].

Many articles disclose minor amendments in the mechanical system but suggest new or improved AFM control and imaging algorithms [105,106,107,108]. Additional control functions are generally implemented which compensate for positioning errors. Usually, the tapping mode is maintained by fast Fourier transformation. However, it was shown that wavelet transformation can also be used for the same purpose [109]. Switching to a different algorithm allows the extraction of the frequency change with selective sensitivity to vibration spectrum components during tip–sample interaction. Wavelet analysis enables one to find the Hamaker constant, which indicates the height of the water layer on the sample surface. Additionally, with the knowledge of the tip radius, the elastic modulus of the sample can be measured.

A systematic summary of the research focused on improvements in scanner design and control algorithms is provided in Table 2.

## 5. Discussion

**Figure 11:**
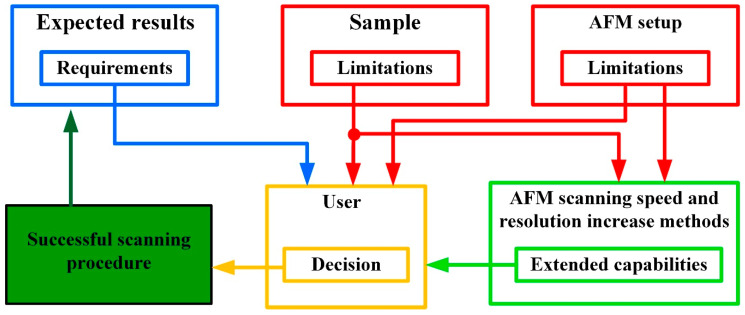
Decision tree of AFM parametrization by sample properties and cantilever parameters optimized for improved performance.

According to the information gathered in the present review, we produced a graphical algorithm of the parametrization of AFM by sample properties and cantilever parameters optimized for improved performance, as illustrated in Figure 11. The graph shows the existing choice tree with a proper solution as output for available initial parameters. The essential input parameters are separated into three parts: the expected results define the requirements for AFM hardware and software; the sample determines the proper scanning mode, influencing AFM parameters; and the AFM setup, including cantilever choice, loops back to the possible quality of the expected image. To avoid the looping process in the user choice procedure, we use restraints in the form of requirements and limitations. Unfortunately, the existing analysis considers no universal methods for AFM parameters nor cantilever characteristics for any current sample. Even with a powerful way of controlling AFM characteristics, the ability to obtain an image within a reasonable time and of good quality with the required resolution and accuracy of a particular sample still depends on the user. Improving the AFM hardware and software of industrially made devices is tricky, as research on technology improvements is usually carried out on AFM setups, which are made in-house and are hardly accepted by the industry.

The selection of the proper AFM scanning mode is a matter of trial and error and not a straightforward decision. For example, it is widely known that the contact mode gives the highest accuracy and allows the fastest scanning. However, there is still uncertainty related to the surface properties of the sample. This mode is unsuitable for soft materials, and there are no well-established methods to determine whether a material is too soft for the contact mode before the actual contact mode scan is performed. Furthermore, it depends on sample stiffness and homogeneity (for example, stiff particles attached to soft base material). Also, the possibility of damaging or unintentionally modifying the sample surface depends on the scanning mode selection and the selection of the cantilever and scanning speed.

Selection between noncontact and tapping modes is not trivial either; both methods have similar imaging capabilities but provide results with different accuracies and differ in the scanning process duration. Choosing a suitable cantilever for each scanning mode is one of the most critical factors for a successful AFM operation. It depends on sample characteristic requirements for expected results and usually relies on the availability of an adequate cantilever and user experience. The decision about cantilever selection can be facilitated using manufacturer recommendations or predictive mathematical models. However, even using a particular model, complex operations can require performing a test scan to evaluate the quality of the obtainable results.

It is essential to note that selecting the proper scanning mode and optimal cantilever parameters remains complicated due to controversy over the technical capabilities of AFM, the quality of results, and scanning duration. It is always necessary to match the required scanning accuracy, image resolution, reliability of results, and scanning time. A higher speed minimizes the needed course of the operation, but at the same time it can reduce the accuracy and reliability of the results. Imaging resolution is also an equally important parameter. Typically, extended imaging time is required for better resolution; nevertheless, it can be balanced out by increasing the scanning speed within accuracy tolerance.

While summarizing the reviewed imaging speed increase methods, we must note that the practical implementation of any method mainly depends on the nature and type of AFM design. Most improvements based on the optimization of cantilever design fit all commercial AFMs since they do not require any physical changes to the AFM structure. On the other hand, cantilevers with perfect functional parameters may lose their benefits due to the limitations of other AFM components. The improvement based on the change of AFM structure or in situ adjustment of the cantilever dynamics requires physical intervention. It is better suited for open AFM designs than for commercial ones. Software scanning pattern optimization and advanced imaging algorithms are the most applicable options for commercial AFMs since they do not require physical intervention into AFM hardware. Such improvements do not conflict with the warranty provided by the manufacturer and can be installed by the potential user according to their specific requirements. Moreover, AFM software improvements can be enhanced by modified cantilevers. It is likely that using such modifications and implementing machine learning algorithms for AFM control and data processing will enable multicriteria modernization of available and new AFMs, increase scanning speed and resolution, and even expand the functionality of AFMs.

## 6. Conclusions

Many new methods and improvements were tested and demonstrated, resulting in a significant increase in scanning speed, up to 400 mm/s in particular cases. Regarding the future prospects of AFM technology, it can reach a more comprehensive implementation, especially in power electronics as a quality control method. This methodology fits the quality control of smart coatings and allows efficient imaging of biological and biochemical items. The faster AFM scanning process will increase technological parameters and foster breakthroughs in nanotechnology and related areas.

AFM capabilities and functionality are developing quite dynamically, as is the popularity of the combination of AFM with other imaging techniques to perform simultaneous multimodal analysis of samples.

## Figures and Tables

**Table 1:** Designs and parameters of cantilevers.

Dimensions ^1^			Spring Constant, N/m	Resonant Frequency, kHz	Working Environment	Ref.
l, μm	w, μm	t, nm				
100–450	50	2000	-	-	Air	[79]
750	160	-	-	48.5	Air	[80]
200 20 85	20 0.75 18	600	(0.1–10) ^−6^	(0.1–100) × 10^3^	Liquid	[55]
5	2	50	0.1	2.8 × 10^3^	-	[56]
23–203	-	-	1.3	-	Air and liquid	[58]
20–400	4–6	600	6.5 × 10^−6^	1.7	-	[60]
10–14	3–5	100	0.1–0.2	100–200	Liquid	[61]
-	-	-	1.7 ± 0.2	61.85 ± 0.1	Liquid	[52]
∼200 ∼388	-	-	0.02–0.03	0–100	Air and liquid	[53]
10–100	-	-	0.01–100	30–190	-	[54]
-	-	-	0.06	-	-	[69]
21 200	0.75 20	- 600	0.5	7–62 35–136	Liquid Air	[66]
200	10–50	1000	-	40–140	Air	[67]
225	27	2700	1.6	61	-	[68]
100 25	30 10	10 2.5	6 74	1690 337	Air and liquid	[81]

^1^ l—length, w—width, t—φthickness.

**Table 2:** AFM scanner design.

Scanning Mode	Improvement	Scanning Area, µm^2^	Achieved Velocities, mm/s	Ref.
Contact mode	Increased scanner stiffness, designed piezoelectric scanner based on the parallel mechanism with flexible links	8 × 7	2	[100]
-	Multiple drives connected in series	120 × 120	3	[101]
Contact mode	Implementation of a resonance-based drive into the scanner *x*-axis	170 × 170	200–440	[110]
Contact mode	Implementation of rotational scanning trajectory	∅ 141.9	45	[102]
Tapping mode	Photothermal cantilever drive and imaging algorithm which considers the delay of system components	1.6 × 1.6	3.2	[103]
Contact mode	Cantilever deflection measurement system based on the optical pickup unit	14 × 14	10	[11]
Contact mode	Implementation of high-speed vertical positioning sinusoidal scanning and high-speed image acquisition	20 × 20	4	[105]
Tapping mode	Analyzed and implemented control techniques based on a modification of *z*-axis feedback and active control of the cantilever quality factor to increase scanning speed and minimize image artifacts	-	-	[106]
-	Increased scanner stiffness and implemented a mechanism with flexible links, enabling the decoupling of separate axes. Implemented the scanner dynamic compensation function into the control algorithm	13 × 13	101.5	[107]
-	Designed a controller for a two-degrees-of-freedom system in which a model-based control method was realized instead of the traditional proportional–integral method	15 × 15	0.15	[108]
-	Designed scanner with optical position measuring system based on interferometers	500 × 80	8	[104]

## Data Availability

The data presented in this study are available on request from the corresponding author. The data are not publicly available due to privacy requirements.
